# Macular retinal circulation in healthy eyes examined by optical coherence tomography angiography extended interscan time analysis

**DOI:** 10.1371/journal.pone.0289896

**Published:** 2023-09-14

**Authors:** Masaharu Ishikura, Yuki Muraoka, Naomi Nishigori, Shin Kadomoto, Shogo Numa, Tomoaki Murakami, Masayuki Hata, Akitaka Tsujikawa

**Affiliations:** Department of Ophthalmology and Visual Sciences, Kyoto University Graduate School of Medicine, Kyoto, Japan; Seirei Hamamatsu General Hospital, JAPAN

## Abstract

**Purpose:**

To examine whether extended interscan time (IST) on optical coherence tomography angiography (OCTA) can detect slow retinal blood flow, which is undetectable on default IST, in the healthy macula.

**Methods:**

OCTA (OCT-A1, Canon Inc.) scanning of a macular area measuring 4 × 4 mm^2^ of 14 healthy eyes of 14 healthy volunteers with no history or evidence of systemic and macular diseases was performed. ISTs were set at 7.6 (IST_7.6_, default setting), 12.0 (IST_12.0_), and 20.6 msec (IST_20.6_). Ten OCTA images were acquired at each IST, and an averaged image was created. For each averaged OCTA image obtained at IST_7.6_, IST_12.0_, and IST_20.6_, we defined the area surrounded by the innermost capillary ring as the foveal avascular zone (FAZ). We qualitatively evaluated the delineation of the capillaries consisting of the FAZ and quantitatively measured the FAZ area at each IST.

**Results:**

Extensions from IST_7.6_ to IST_12.0_ and IST_20.6_ could newly delineated retinal capillaries that were undetectable at the default IST; new capillaries were detected in 10 (71%) eyes at IST_12.0_ and 11 (78%) eyes at IST_20.0_. The FAZ areas were 0.334 ± 0.137 mm^2^, 0.320 ± 0.132 mm^2^, and 0.319 ± 0.129 mm^2^ for IST_7.6_, IST_12.0_, and IST_20.0_, respectively; the FAZ areas at IST_12.0_ and IST_20.0_ were significantly decreased compared with that at IST_7.6_ (*p* = 0.004 and 0.002, respectively).

**Conclusion:**

In OCTA for healthy participants, extensions of the ISTs newly detected retinal capillaries with slow blood flow around FAZ. The FAZ shapes varied with different ISTs. Thus, the blood flow dynamics are not physiologically uniform around FAZ. Compared with conventional OCTA, this protocol enables a more detailed evaluation of retinal circulation and provides a better understanding of the physiological circulatory status of the healthy retina, and may enable the assessment of circulation in the very early stages in diseased eyes.

## Introduction

Fluorescein angiography (FA) and indocyanine green angiography (ICGA) are imaging modalities used for visualizing the retinal microvasculature [1, 2]. FA/ICGA can visualize lesions with increased vascular permeability, no or hypoperfusion, and neovascularization and are particularly important in the diagnosis and monitoring of various diseases, such as diabetic retinopathy (DR), retinal arterial- and vein occlusions (RVO), and age-related macular degeneration. However, these angiography modalities are somewhat invasive as they require the intravenous injection of dyes and are not easy to perform frequently [3, 4].

Optical coherence tomography angiography (OCTA) is an imaging modality that can be performed noninvasively and easily without dye injections and evaluate the detailed vascular lesion in three dimensions [5]. The OCTA images are generated by repeatedly scanning the same location and analyzing the changes in the intensity and phase between the interscan time (IST). The blood flow (erythrocyte dynamics) in the retinal vessels changes dynamically with time. OCTA depicts the decorrelation signal from the erythrocytes beyond a certain threshold at the default IST setting [6]. However, capillaries with erythrocyte velocities below a certain threshold cannot be depicted as the distance of the erythrocyte movement between ISTs is inferior to the resolution of OCTA [7–10].

The blood flow in the capillaries that form the foveal avascular zone (FAZ) is prone to stagnation for some reason in common retinal circulatory diseases, such as DR and RVO, and FAZ tends to enlarge. FAZ enlargement is considered an important clinical marker because of its significant associations with disease severity [11, 12]. However, its characteristics are not well understood in diseased eyes. Furthermore, the hemodynamics around FAZ remains to be fully investigated in healthy eyes.

Recently, a methodology, called variable IST analysis (VISTA), was developed to detect abnormal blood flow in OCTA by changing the ISTs [13]. Novel findings regarding DR and RVO have been reported using VISTA [14–17]. In this study, which included healthy participants, we examined the retinal circulation status at the macula using multiple IST, similar to that in VISTA.

## Materials and methods

### Participants and methods

This observational study was approved by the Institutional Review Board of the Kyoto University Graduate School of Medicine (Kyoto, Japan) and adhered to the tenets of the Declaration of Helsinki. Written informed consent was obtained from each participant before initiating any study procedures or examinations.

We included 14 healthy eyes of 14 healthy volunteers with no history or evidence of systemic and macula diseases (Table 1). The mean age of the participants was 44 ± 17 years (range, 25–73 years).

**Table 1:** Characteristics of healthy participants.

Number (men/women)	14 (9/5)
Age (range), years	44 ± 17, (25–73)
Best-corrected visual acuity in logMAR	-0.15 ± 0.05
Snellen equivalent (range)	20/20–20/13
Intraocular pressure, mmHg	14.9 ± 2.2
Axial length, mm	24.93 ± 0.90
Systolic blood pressure, mmHg	117 ± 16
Diastolic blood pressure, mmHg	73 ± 11

Data are presented as the mean ± standard deviation unless otherwise indicated.

logMAR, logarithm of the minimal angle of resolution.

We excluded eyes with ocular diseases such as keratoconus, high myopia (severity exceeding −6 diopters), and/or high astigmatism (severity exceeding ±3 diopters). We also excluded eyes with poor-quality OCTA images (signal strength index < 6) caused by eye movement or media opacities.

### OCTA using multiple ISTs

At the time of inclusion in the study, each participant underwent high-resolution spectral-domain OCTA (OCT-A1, Canon Inc., Tokyo, Japan; OCT beam spot size on the retina is 20 μm) in addition to routine examinations, including the measurement of the best-corrected visual acuity (BCVA) using a Landolt chart, measurement of the intraocular pressure using the non-contact tonometor (NT-530P NIDEK CO., LTD. Aichi, Japan), the axial length was measured using an interferometer (IOL Master 700; Carl Zeiss Meditec, La Jolla, CA, USA), slit-lamp biomicroscopy, and OCT. In all cases, the subjects had their resting blood pressure measured approximately 30 min before OCTA was performed. All examinations were performed on the same day.

For OCTA images, a macular area measuring 4 × 4 mm^2^ (464 × 464 pixels) was scanned using ISTs of 7.6 msec (IST_7.6_), 12.0 msec (IST_12.0_), and 20.6 msec (IST_20.6_) in a repeated mode, which enables capturing the same area when the ISTs are changed. The participants underwent imaging ten times at each IST, and the averaged OCTA images were created. All OCTA images were acquired at the same resolution to eliminate differences in imaging conditions other than IST. The region from the inner limiting membrane to the inner segment-outer segment line, which included all capillaries in the inner retina, was targeted for evaluation to ensure that segmentation would not affect the depiction of blood vessels.

### Definition of FAZ and capillaries for hemodynamic analysis

We defined FAZ on an OCTA image at each IST using the FAZ measuring tool of the software (Canon Inc.), which automatically determined FAZ as the area without blood vessels in the macula. Additionally, we qualitatively evaluated the capillaries with the high-intensity area comprising the contour of FAZ.

While measuring the FAZ area, we unified the measurement range among the participants by correcting the AL-related magnification using the modified Littmann formula (Bennett procedure) [18–20]. The area ratio to be corrected was calculated by referring to the axial length of each participant’s eye and squaring the length expansion factor calculated using Bennett’s formula (1).


$$Area\;ratio\;to\;be\;corrected\;={\left(AL-1.82\right)}^{2}/\;{\left(standard\;AL-1.82\right)}^{2}$$ (1)

For OCT-A1, the standard AL provided by the manufacturer was set to 24.20 mm. In each case, the FAZ area was multiplied by the area ratio to derive the axial-length-corrected FAZ area.

### Statistical analysis

Statistical analyses were performed using JMP 16 (SAS Institute Inc., Cary, NC, USA). All values are presented as the mean ± standard deviation. We converted the decimal BCVA measured using a Landolt chart to a logarithm of the minimum angle of resolution (logMAR).

The FAZ areas captured at IST_7.6_, IST_12.0_, and IST_20.6_ were analyzed using the Shapiro–Wilk test to evaluate the distribution of normality. Statistical significance was set at *p*<0.05. The FAZ areas captured at all ISTs were within the distribution of normality. Comparisons between the FAZ areas captured at IST_7.6_ and those captured at IST_12.0_ and between the FAZ areas captured at IST_7.6_ and those captured at IST_20.6_ were performed using the paired t-test.

## Results

Table 1 shows the characteristics of the participants. No visual disturbances were observed in any of the participants. The mean logMAR BCVA was -0.15 ± 0.05, the intraocular pressure was 14.9 ± 2.2 mmHg, the axial length was 24.93 ± 0.90, and the systolic and diastolic blood pressures were 117 ± 16 mmHg and 73 ± 11 mmHg, respectively.

### OCTA images of the macula in healthy eyes obtained using multiple ISTs

**Fig 1:**
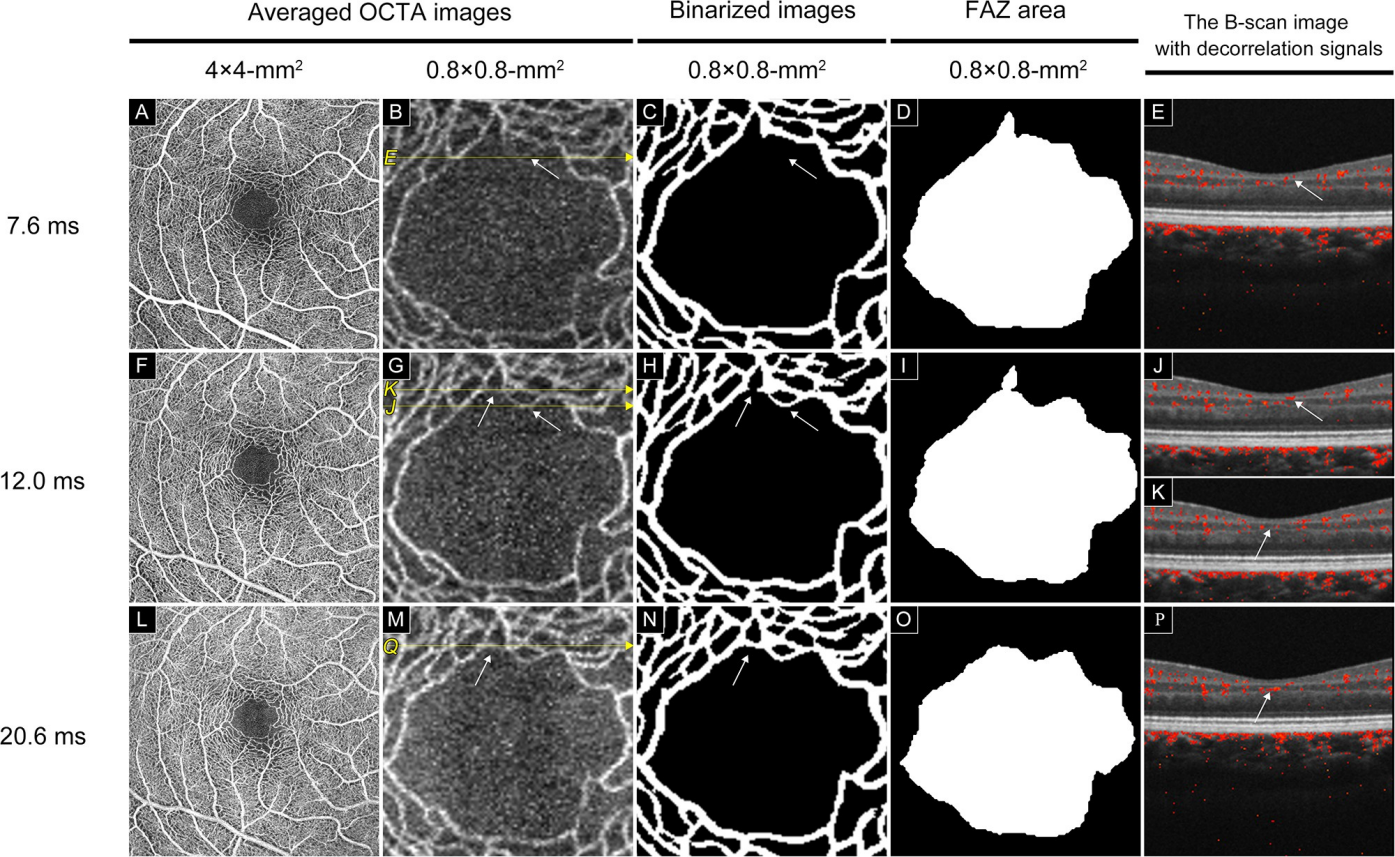
Capillaries are depicted more clearly with extension (IST_7.6_, IST_12.0_, and IST_20_) of the interscan time; OCTA images of the healthy macular area in the healthy eye. A, The averaged OCTA image of a 4 × 4 mm^2^ macular area created by averaging ten OCTA images obtained at IST_7.6_. B, The averaged OCTA image of a 0.8 × 0.8 mm^2^ area around the FAZ magnified from image A. C, Binarized image of the capillaries from image B created using the software made by Canon Inc. D, The area of FAZ obtained from image C. E, The OCT B-scan image and decorrelation signal of B. No decorrelation signal is observed (white arrow). F, The averaged OCTA image of a 4 × 4 mm^2^ macular area created by averaging ten OCTA images obtained at IST_12.0_. G, The averaged OCTA image of a 0.8 × 0.8 mm^2^ area around the FAZ magnified from image E. H, Binarized image of the capillaries from image F created using the software made by Canon Inc. An additional capillary is depicted in image G, compared with the OCTA image obtained at IST_7.6_ (White arrow). I, The area of FAZ obtained from image G. J: The OCT B-scan image and decorrelation signal of G. A decorrelation signal not depicted in E is observed (white arrow). K The OCT B-scan image and G decorrelation signal: No decorrelation signal is confirmed (white arrow). The averaged OCTA image of a 4 × 4 mm^2^ macular area was created by averaging ten OCTA images obtained at IST_20.6_. M, The averaged OCTA image of a 0.8 × 0.8 mm^2^ area around FAZ magnified from image I. N, A binarized image of the capillaries from image J is created using software made by Canon Inc. An additional capillary is depicted in image G compared with the OCTA images obtained at IST_7.6_ and IST_12.0_ (White arrow). O, The area of FAZ obtained from image K. P, The OCT B-scan image and decorrelation signal of M. A decorrelation signal not depicted in E is observed (white arrow).

**Fig 2:**
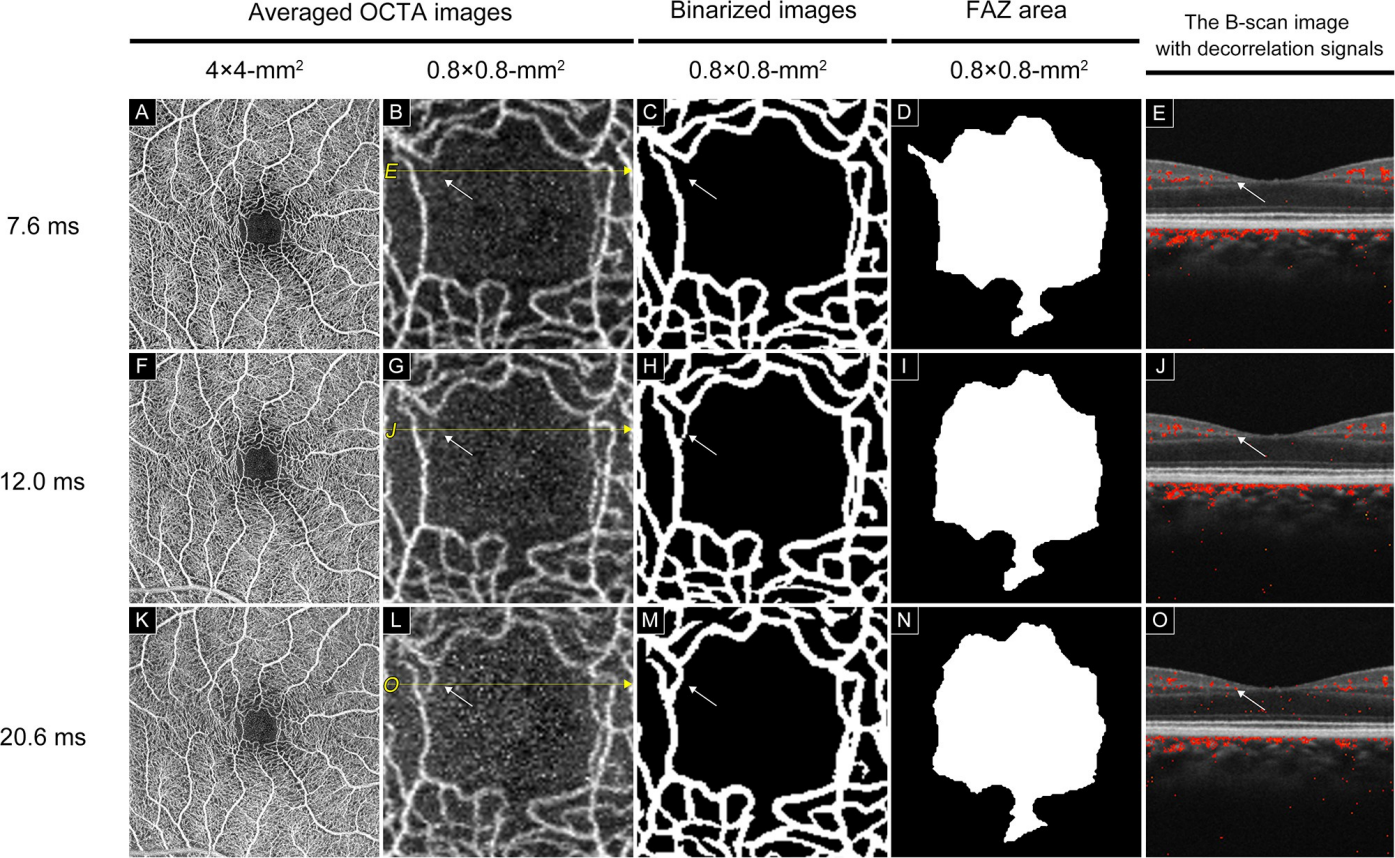
Capillaries become more intense with each extended interscan time (IST_7.6_, IST_12.0_, and IST_20.6_); OCTA images of the healthy macular area in the healthy eye. A, The averaged OCTA image of a 4 × 4 mm^2^ macular area created by averaging ten OCTA images obtained at IST_7.6_. B, The averaged OCTA image of a 0.8 × 0.8 mm^2^ area around the FAZ magnified from image A. C, Binarized image of the capillaries from image B created using the software made by Canon Inc. D, The area of FAZ obtained from image C. E, The OCT B-scan image and decorrelation signal of B. No decorrelation signal is observed (white arrow). F, The averaged OCTA image of a 4 × 4 mm^2^ macular area created by averaging ten OCTA images obtained at IST_12.0_. G, The averaged OCTA image of a 0.8 × 0.8 mm^2^ area around the FAZ magnified from image E. The capillary is more intense at IST_12.0_ than that at IST_7.6_ (White arrow). H, Binarized image of the capillaries from image F created using the software made by Canon, Inc. I, The area of FAZ obtained from image G. J: The OCT B-scan image and decorrelation signal of G. A decorrelation signal not depicted in E is observed (white arrow). The averaged OCTA image of a 4 × 4 mm^2^ macular area is created by averaging ten OCTA images obtained at IST_20.6_. L, The averaged OCTA image of a 0.8 × 0.8 mm^2^ area around FAZ magnified from image I. The capillaries are more intense at IST_20.6_ than that at IST_12.0_ (White arrows). M, A binarized image of capillaries from image J is created using software made by Canon Inc. N, The area of FAZ obtained from image K. O The OCT B-scan image and L decorrelation signal The decorrelation signal increased compared to that in E and J (white arrow).

Extensions of the IST_7.6_ (default setting) to IST_12.0_ and IST_20.6_ could newly detect retinal capillaries that were undetectable at the default IST_7.6_ (Figs 1 and 2); new capillaries were detected in 10 (71%) eyes at IST_12.0_ and 11 (78%) eyes at IST_20.0_.

### FAZ area obtained by OCTA using multiple ISTs

**Fig 3:**
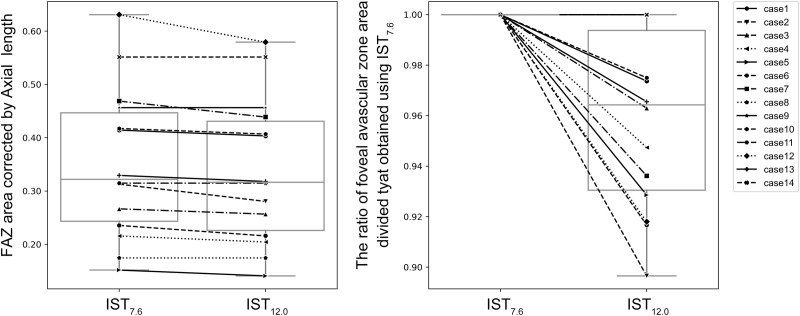
Comparison of the FAZ areas obtained at IST_7.6_ and IST_12.0._ In the graph on the left, the vertical axis represents the FAZ area corrected by the axial length obtained at IST_7.6_ and IST_12.0_, whereas the horizontal axis represents IST. In the graph on the right, the vertical axis represents the area of FAZ obtained at IST_7.6_ and IST_12.0_ divided by the area of FAZ obtained at IST_7.6_; the horizontal axis represents IST. The FAZ areas decreased significantly at IST_12.0_ compared with that at IST_7.6_.

**Fig 4:**
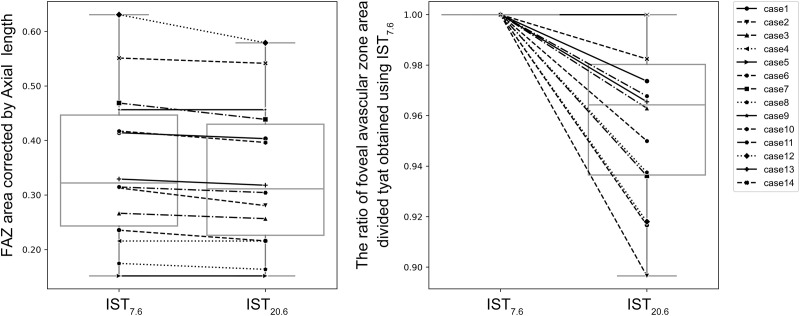
Comparison of FAZ areas obtained at IST_7.6_ and IST_20.6._ In the graph on the left, the vertical axis represents the FAZ area corrected by the axial length obtained at IST_7.6_ and IST_20.6_, whereas the horizontal axis represents IST. In the graph on the right, the vertical axis represents the area of FAZ obtained at IST_7.6_ and IST_20.6_ divided by the area of FAZ obtained at IST_7.6_; the horizontal axis represents IST. The FAZ areas decreased significantly at IST_20.6_ compared with that at IST_7.6_.

The FAZ areas were 0.334 ± 0.137 mm^2^ for IST_7.6_, 0.320 ± 0.132 mm^2^ for IST_12.0_, and 0.319 ± 0.129 mm^2^ for IST_20.0_; The axial length corrected FAZ areas were 0.353 ± 0.137 mm^2^ for IST_7.6_, 0.339 ± 0.133 mm^2^ for IST_12.0_, and 0.337 ± 0.130 mm^2^ for IST_20.0_; the axial length corrected FAZ areas decreased significantly at IST_12.0_ and IST_20.0_ compared with that at IST_7.6_ (*p* = 0.004 and 0.002, respectively) (Figs 3 and 4). The ratios of the FAZ areas captured at IST_12.0_ and IST_20.0_ compared with that at IST_7.6_ were 0.96 ± 0.03 and 0.96 ± 0.03, respectively (Figs 3 and 4).

## Discussion

OCTA was performed using multiple ISTs in this study. Three different ISTs were set to analyze the details of the macular retinal circulation in healthy eyes. The extensions of the IST_7.6_ to IST_12.0_ and IST_20.6_ resulted in the depiction of new capillaries around FAZ, and the FAZ areas were found to decrease significantly (Figs 1–4).

In the era before the advent of OCTA, FA was the standard imaging modality used for evaluating the retinal circulatory status. FA has the advantage of enabling the evaluation of fluorescence leakage, which is not possible with OCTA. However, FA is invasive as it requires the intravenous injection of dyes and is not easy to perform frequently [3]. In contrast, OCTA has the advantages of shorter examination time and high resolution with three-dimensional images; moreover, it is non-invasive as the use of dyes is not required. Thus, OCTA has become the leading modality for the evaluation of the retinal circulatory status.

In OCTA, images are generated by analyzing multiple repetitive B-scans performed at the same location. The principle of OCTA is to image the changes in the intensity and phase of the B-scans obtained via repeated scans, and any change in the image during IST is imaged as blood flow. Clinically, OCTA is usually performed with a single IST. Various pathologic changes in the retinal vasculatures have been reported using OCTA, such as the enlargement of NPA [17] and the classification of MA [8, 21]. The enlarged FAZs of eyes with DR [22, 23] and RVO [24] were shown to be associated with a decrease in visual acuity, and evaluating FAZ is considered to be useful for assessing the severity of these retinal circulatory diseases [25]. However, the reasons underlying the enlargement of FAZ with the progression of the disease severity are not well understood.

Ploner et al. recently developed a VISTA protocol, in which they shortened the default IST of 3.0 msec to 1.5 msec, and found changes in the retinal blood flow velocities in diseased eyes with DR, geographic atrophy, and exudative age-related macular degeneration [13]. Using the same VISTA protocol, the changes were observed in the blood flow of choriocapillaris or polypoidal lesions in eyes with GA [26] and polypoidal choroidal vasculopathy [27]. Previous reports have shown that analyzing OCTA images with ISTs of 1.5 and 3.0 msec extends the range of blood flow velocities that can be analyzed compared with that obtained with a single IST. In this study, we extended the default IST of 7.6 msec to 12.0 msec and 20.6 msec to effectively detect slow blood flow, which differed from the strategy of shortening the IST used in previous studies [13, 26, 27]. This was a novel attempt to examine whether the length of conventional ISTs is sufficient to capture all retinal capillary blood flow.

The protocol used in this study showed that the retinal capillary circulation around FAZ was not uniform in healthy eyes (Figs 3 and 4). The OCTA decorrelation signal is obtained when the OCT signal in the capillaries changes beyond a threshold value during the time between each scan.

The blood flow velocities in capillaries have been reported previously using adaptive optical scanning laser ophthalmoscope (AOSLO). The capillaries that required extended IST in this study to be depicted would have a slower velocity [28]. A previous AOSLO study visualized leukocytes and erythrocytes passing through the retinal capillaries of healthy subjects and suggested that leukocyte movement could generate changes in local pressure within the capillaries and that the blood velocity in and around the capillaries might be altered [28]. This may explain the variation in the retinal blood flow velocities around FAZ in this study.

Compared with healthy eyes, the retinal blood flow in diseased eyes tends to be deteriorated [13, 14]. Nishigori et al. successfully detected retinal capillaries with congestive slow blood flow that were associated with the future recurrence of macular edema from branch RVO using a VISTA protocol similar to that used in this study [15]. The results of this study suggested that there might be a physiological slowing of retinal blood flow in the healthy macula that required an extension of the IST to be detected. Such capillaries may be more likely to be more susceptible to retinal circulatory disturbance than the capillaries that did not require prolonged IST to be detected.

This study has several limitations. First, this study was a cross-sectional study, and longitudinal changes in the retinal circulatory status were not observed. Second, we used three ISTs in the analysis, and the results of other ISTs are unknown. Third, the number of eyes included was limited. The protocol in this study was applied for examining the region around FAZ, and the retinal circulatory status in other outer retinal areas was unknown.

Nevertheless, the extensions of ISTs newly detected retinal capillaries with slow blood flow around FAZs of healthy eyes, and the FAZ shapes changed as the ISTs varied. These findings indicate that the retinal circulation is not physiologically uniform around FAZ. The protocol of this study may have the potential to enable a detailed assessment of the circulatory status in healthy eyes and eyes with retinal diseases. Further prospective studies with a greater number of participants and pathologic eyes are required to confirm the findings of the present study.
